# Readmission Risk Trajectories for Patients With Heart Failure Using a Dynamic Prediction Approach: Retrospective Study

**DOI:** 10.2196/14756

**Published:** 2019-09-16

**Authors:** Wei Jiang, Sauleh Siddiqui, Sean Barnes, Lili A Barouch, Frederick Korley, Diego A Martinez, Matthew Toerper, Stephanie Cabral, Eric Hamrock, Scott Levin

**Affiliations:** 1 Department of Civil Engineering, Johns Hopkins System Institute Johns Hopkins University Baltimore, MD United States; 2 Department of Decision, Operations & Information Technologies, Robert H Smith School of Business University of Maryland College Park, MD United States; 3 Department of Medicine, Division of Cardiology Johns Hopkins University School of Medicine Baltimore, MD United States; 4 Department of Emergency Medicine University of Michigan Ann Arbor, MI United States; 5 Department of Emergency Medicine Johns Hopkins University School of Medicine Baltimore, MD United States; 6 Department of Epidemiology & Public Health University of Maryland College Park, MD United States; 7 Innovation and Continuous Improvement Department Howard County General Hospital Columbia, MD United States; 8 StoCastic, LLC Towson, MD United States

**Keywords:** heart failure, patient readmission, forecasting, machine learning

## Abstract

**Background:**

Patients hospitalized with heart failure suffer the highest rates of 30-day readmission among other clinically defined patient populations in the United States. Investigation into the predictability of 30-day readmissions can lead to clinical decision support tools and targeted interventions that can help care providers to improve individual patient care and reduce readmission risk.

**Objective:**

This study aimed to develop a dynamic readmission risk prediction model that yields daily predictions for patients hospitalized with heart failure toward identifying risk trajectories over time and identifying clinical predictors associated with different patterns in readmission risk trajectories.

**Methods:**

A two-stage predictive modeling approach combining logistic and beta regression was applied to electronic health record data accumulated daily to predict 30-day readmission for 534 hospital encounters of patients with heart failure over 2750 patient days. Unsupervised clustering was performed on predictions to uncover time-dependent trends in readmission risk over the patient’s hospital stay. We used data collected between September 1, 2013, and August 31, 2015, from a community hospital in Maryland (United States) for patients with a primary diagnosis of heart failure. Patients who died during the hospital stay or were transferred to other acute care hospitals or hospice care were excluded.

**Results:**

Readmission occurred in 107 (107/534, 20.0%) encounters. The out-of-sample area under curve for the 2-stage predictive model was 0.73 (SD 0.08). Dynamic clinical predictors capturing laboratory results and vital signs had the highest predictive value compared with demographic, administrative, medical, and procedural data included. Unsupervised clustering identified four risk trajectory groups: decreasing risk (131/534, 24.5% encounters), high risk (113/534, 21.2%), moderate risk (177/534, 33.1%), and low risk (113/534, 21.2%). The decreasing risk group demonstrated change in average probability of readmission from admission (0.69) to discharge (0.30), whereas the high risk (0.75), moderate risk (0.61), and low risk (0.39) groups maintained consistency over the hospital course. A higher level of hemoglobin, larger decrease in potassium and diastolic blood pressure from admission to discharge, and smaller number of past hospitalizations are associated with decreasing readmission risk (*P*<.001).

**Conclusions:**

Dynamically predicting readmission and quantifying trends over patients’ hospital stay illuminated differing risk trajectory groups. Identifying risk trajectory patterns and distinguishing predictors may shed new light on indicators of readmission and the isolated effects of the index hospitalization.

## Introduction

### Background

Patients hospitalized with heart failure suffer the highest rates of 30-day readmission among other clinically defined patient populations in the United States [1]. National efforts to prevent avoidable hospitalizations have led to the adoption of 30-day readmission as a publicly reported performance measure linked to Medicare patient reimbursement [2]. This has motivated much investigation into the predictability of 30-day in-hospital readmissions to guide targeted interventions that could reduce risk. These investigations can lead to clinical decision support tools that can be made available to care providers to guide them in individual patient care. Clinical and administrative data available in-hospital electronic health records (EHRs) and clinical registries have been the primary data sources for these evaluations. Previous studies have applied traditional statistical- and machine learning–based methods for predicting 30-day readmission with varied success for cohorts of patients with heart failure; predictive performance measured as the area under the receiver operating characteristic curve (area under curve [AUC]) has ranged from 0.55 to 0.76 [3-8].

These past readmission prediction models were static—designed to function at a single point in time—either at the beginning of the patient’s stay at admission where data are limited [3] or near discharge where all clinical and administrative data may become available for each patient [4-7]. Readmission predictions that occur at admission are useful in creating awareness of risk early in the hospitalization to trigger interventions that may be performed in-hospital and allow for anticipation of postacute care needs. However, these models tend to have lower accuracy because of the lack of availability of meaningful clinical data for prediction. Alternatively, readmission models that operate near discharge are more accurate but may be more difficult to integrate into the workflow and limit the time to perform any in-hospital–based interventions. In either case, these predictive models are static, not designed to determine how readmission risk may fluctuate as a result of clinical findings and hospital interventions. As a result, they do not lend themselves to be enhanced into dynamic decision support tools and are often not applied in general hospital settings. The motivation for this study rests on developing a tool that can eventually be used by care providers to assist in medical decision making. The study is a first step in developing an implementation toward decision support in treatment for patients with heart failure.

In this study, we dynamically predicted a 30-day readmission risk on each day of the patient’s hospitalization assuming that the patient was discharged on that day. The reason to predict this counterfactual outcome is that, by definition, 30-day readmission is readmission within 30 days after discharge. Therefore, a discharge time must be defined while predicting 30-day readmission risk. The current day is most appropriate as the discharge day for real-time prediction as care providers are most interested in learning the readmission risk if the patient is to be discharged that day. We can then keep updating this risk daily for the duration of stay. This may be able to aid physicians in their decision on when to discharge a patient.

### Objective

The objective of this study was to develop a predictive model of 30-day readmission for patients with heart failure that functions in real time over the course of a patient’s hospitalization. We made predictions for the risk of 30-day readmission as if the patient were discharged on each day of their hospitalization to illustrate how this risk varied over time (if at all) for each patient. We hypothesized that quantifying time-dependent trends in readmission risk had the potential to illuminate the effects of clinical measures and interventions on readmission likelihood at discharge. These predictions could potentially support physician decision making on when to safely discharge patients from the hospital based on the current level of risk and trend over time. These analyses further enabled the identification of groups of patients with heart failure having differing trajectories of readmission risk over their hospital stay.

## Methods

### Setting and Data

We conducted a retrospective cohort study of patients with a primary diagnosis of heart failure between September 1, 2013, and August 31, 2015, from a community hospital in Maryland. Patients with heart failure were identified using the International Classification of Diseases–ninth revision codes: 428.x, 402.01, 402.11, 402.91, 404.01, 404.03, 404.11, 404.13, 404.91, and 404.93 [9,10]. Patients who died during the hospital stay or were transferred to other acute care hospitals or hospice care were excluded. The final cohort consisted of 534 encounters totaling 2750 patient days (median: 4 days; interquartile range: 4 days). Data collected as part of routine patient care were extracted from the hospital EHR system for our readmission outcome and predictor variables.

As per the Centers for Medicare and Medicaid Services definition, we defined our primary outcome 30-day readmission as all-cause readmission to the same hospital within 30 days after discharge from the index hospitalization [2].

We derived hypothesized predictors from heart failure and readmission risk factors used in previous studies as summarized in Table 1 [5,11-16]. Predictor data may be conceptually grouped into static (unchanged over the hospital stay) or dynamic (fluctuating over the hospital stay) categories. Static predictors comprised demographics (eg, age, gender, and race), socioeconomic status (eg, insurance, marital status, and ZIP code), health care utilization (eg, discharge disposition and number of visits in the last 6 months), and clinically categorized chief complaint [17,18]. Charlson comorbidity index at admission was computed using past medical history (ie, active problems) [12,13] and admission diagnoses.

**Table 1:** Descriptive summary of patient characteristics for each 30-day readmission outcome.

Static predictors		Not readmitted (n=427)	Readmitted (n=107)	*P* value
Age (years), mean, median (IQR^a^)		74.5, 77 (66-85)	75.2, 78 (67-87)	.37
Gender: female, n (%)		223 (52.2)	64 (59.8)	.19
**Marital status, n (%)**				.81
	Married	168 (39.3)	44 (41.1)	
	Single	68 (15.9)	19 (17.8)	
	Widowed	147 (34.4)	36 (33.6)	
**Race, n (%)**				.96
	White	258 (60.4)	65 (60.7)	
	Black or African American	121 (28.3)	31 (29.0)	
	Asian	36 (8.4)	9 (8.4)	
**Insurance, n (%)**				.46
	Medicare	300 (70.2)	80 (74.8)	
	Commercial	92 (21.5)	21 (19.6)	
	Medicaid	13 (3.0)	4 (3.7)	
	Other	22 (5.2)	2 (1.9)	
**Discharge disposition, n (%)**				.35
	Home or self-care	276 (64.6)	65 (60.7)	
	Skilled nursing facility	62 (14.5)	13 (12.1)	
	Home–health care service	49 (11.5)	16 (15.0)	
	Rehabilitation facility	17 (4.0)	6 (5.6)	
	Short-term hospital	8 (1.9)	2 (1.9)	
	Nursing facility	5 (1.2)	5 (4.7)	
Number of past visits to hospital, mean, median (IQR^a^)		1.4, 1 (0-2)	2.2, 1 (0-3)	.002
**Chief complaints, n (%)**				.91
	Shortness of breath	282 (66.0)	64 (59.8)	
	Chest pain	26 (6.1)	7 (6.5)	
	Edema	17 (4.0)	6 (5.6)	
	Weakness	15 (3.5)	3 (2.8)	
	Lower respiratory tract infection	12 (2.8)	2 (1.9)	
	Abdominal pain	9 (2.1)	3 (2.8)	
	General	7 (1.6)	1 (0.9)	
	Altered mental status	6 (1.4)	1 (0.9)	
	Genitourinary	5 (1.2)	2 (1.9)	
	Blunt trauma	5 (1.2)	3 (2.8)	
ZIP code		—^b^	—	—
Diagnoses		—	—	—
Diagnoses history		—	—	—
**Dynamic predictors**				
	Elapsed length of stay, mean, median (IQR^a^)	4.8, 3.7 (2.2-5.8)	6.4, 4.2 (2.6-7.3)	.02
**Laboratory** **test, mean, median (IQR** ^a^ **)**				
	Alanine transaminase (units/L)	32.0, 20.0 (13.3-30.0)	28.0, 18.0 (12.0-28.6)	.24
	Aspartate transaminase (units/L)	32.7, 24.0 (18.0-35.0)	32.3, 21.8 (16.9-30.0)	.19
	Blood urea nitrogen (mg/dL)	28.5, 25.0 (18.3-35.3)	34.2, 30.8 (20.0-44.7)	.006
	Creatinine (mg/dL)	1.6, 1.2 (1.0-1.6)	1.9, 1.4 (1.0-2.0)	.004
	Hemoglobin (mg/dL)	11.4, 11.2 (9.9-12.7)	10.8, 10.4 (9.1-12.3)	.003
	Potassium (mmol/L)	4.2, 4.1 (3.9-4.4)	4.2, 4.1 (3.9-4.4)	.83
	Sodium (mmol/L)	139.0, 139.0 (137.0-141.5)	137.7, 137.8 (134.8-140.9)	.003
	pro B-type natriuretic peptide (pg/mL)	8182.5, 4283.0 (2137.0-8656.0)	8683.3, 4262.0 (2013.0-9545.0)	.92
	Troponin T (pg/mL)	37.1, 10.0 (10.0-30.0)	56.1, 14.2 (10.0-52.7)	.14
**Vital signs**				
	Systolic blood pressure	—	—	—
	Diastolic blood pressure	—	—	—
	Temperature	—	—	—
	Respiratory rate	—	—	—
	Pulse	—	—	—
	Peripheral capillary oxygen	—	—	—
Weight		—	—	—
Medication order		—	—	—
Procedure		—	—	—

^a^IQR: interquartile range.

^b^Not applicable (indicates categorical predictors with too many values or longitudinal variables with repeated measurements that cannot be easily summarized in a single table cell).

Dynamic predictors comprised variables dependent on the time of prediction and included the elapsed length of stay, vital signs (systolic blood pressure, diastolic blood pressure, temperature, respiratory rate, pulse, and peripheral capillary oxygen), and laboratory results (alanine transaminase [ALT]; aspartate transaminase [AST]; blood urea nitrogen [BUN]; creatinine; hemoglobin; potassium; sodium; pro B-type natriuretic peptide [proBNP]; and troponin T). Time series characteristics of vital signs and laboratory results were engineered as predictors in the following manner:

1. Number of measurements: n.
2. Average value
3. Standard deviation
4. Minimum value
5. Maximum value
6. Normalized index of the minimum: index of minimal value / n
7. Normalized index of the maximum: index of maximal value / n
8. Average of last 3 measurements
9. Average of first-order difference

Normalized index predictors were designed to capture the relative point during hospitalization when these extreme measurements occurred [19]. For example, if 10 temperature measurements have been observed up to a particular prediction time, and the sixth measurement is where the minimum occurred, the value of the normalized index is equal to 6/10=0.6.

All laboratory tests were not necessarily available for each encounter in the database. For example, 70 out of the 534 encounters did not have proBNP measurement data, 55 did not have AST data, and 37 did not have troponin T data. We excluded laboratory-related predictors with more than 30% unavailable data and imputed missing values with the encounter-based mean for features with less than 30% missing data. Predictors quantifying the number of measurements (ie, lab values) were created from the hypothesis that increased measurement intensity might relate to severity of illness and likelihood of readmission [20]. This also enabled inclusion of binary indicators for missing laboratory values which might have meaning.

### Daily Prediction Model

Our goal was to build a daily prediction model structured to yield a prediction of 30-day readmission risk each day (6 am) during the patients’ stay based on data available at the time of prediction. Thus, predictions made earlier in the stay are based on less information than predictions made later. We generated predictions at the time of hospital admission, then 6 am each subsequent day, and finally at hospital discharge.

The challenge for building a daily prediction model is that the daily outcomes are unknown. Thus, for a particular day, we do not observe whether or not a patient would be readmitted if they were discharged earlier or later than their observed discharge date. To address this uncertainty, we used a 2-stage modeling approach. In the first stage, we estimated the daily risk of readmission based on knowledge of the full encounter data. In the second stage, we used this estimated risk as the outcome for daily prediction using only the data available up to that particular prediction time. We used the first-stage predictions as a proxy for the unobservable outcomes in the second stage.

Specifically, in the first stage, we trained a logistic regression model for 30-day readmission based on all of the information available for each encounter at the time of discharge. Backward selection was used to eliminate predictors without predictive value in the first-stage model. Each observation for this training data corresponded to a patient encounter (ie, encounter-level model).

In the subsequent second stage, we trained a beta regression model to predict the daily risk of 30-day readmission based on the risk of readmission predicted (on the same day) by the first-stage model. We call this the *counterfactual readmission risk* as we are estimating this risk assuming that the patients were discharged earlier than their actual observed discharge time. Each observation in the training data for the second-stage model corresponds to a patient day that comprises the cumulative data available up to the prediction time (eg, 6 am). An example of the second-stage training data is shown in Multimedia Appendix 1, which includes the same set of predictors as those used in the first stage. However, these predictors were structured differently to exclusively account for the information available at the daily prediction time. For example, the elapsed length of stay and normalized index of minimal potassium are constant for each encounter in the first-stage training data, but dynamically change for each observation in the second-stage (daily) training data. In particular, consider the situation when the minimal potassium measurement occurred on day 3 for a patient whose length of stay is 6 days. For this patient, the normalized index of minimal potassium would be 0.5 in the first-stage training data and 1 in the second-stage training data for the day 3 prediction.

Readmission risk from the first-stage model output was bounded between 0 and 1 and followed a beta distribution (Kolmogorov-Smirnov goodness-of-fit test *P*>.05) [21]. Thus, beta regression was applied in the second stage to predict daily readmission risk from the first-stage output. For the readmission risk at discharge, the second-stage model used the estimated risk from the first-stage model as a representation of the actual risk of readmission at that point in time.

Both the first-stage and second-stage models were developed using 5-fold cross-validation (427/534, 79.9% training and 107/534, 20.0% test). Predictive performance measures of the AUC for the first-stage model (encounter-level), and pseudo R-squared value for the second-stage model (daily) were evaluated out-of-sample. R-squared represents the squared correlation coefficient between the counterfactual daily readmission risk and predicted values from the beta regression model.

### Patient Risk Trajectory Cluster Analysis

A patient’s daily 30-day readmission risk may change over the course of their hospitalization because of the evolution of their condition as therapies are administered. A total of 78.4% (107,299/136,911) of all laboratory tests, 74.0% (2173/2935) of all procedures, and 92.5% (3937/4258) of all medication orders occurred before the end of day 5 for our study cohort. Thus, we believe that the majority of variation in readmission risk occurred before this time. After training the beta regression model in the second stage, we used the trained model for predicting the daily readmission risk at admission—day 1 to day 5—and discharge. We applied unsupervised clustering using the K-means algorithm to determine readmission risk trajectories over these 7 specific milestones during a patients’ stay [22]. We used this approach to learn potential trajectories (ie, time-dependent trends) in readmission risk that might naturally distinguish patient groups. Predictors differentiating these trajectory groups were then identified using Kruskal-Wallis hypothesis testing [23]. We further investigated how these discriminant predictors changed with time within each patient risk group.

**Figure 1:**
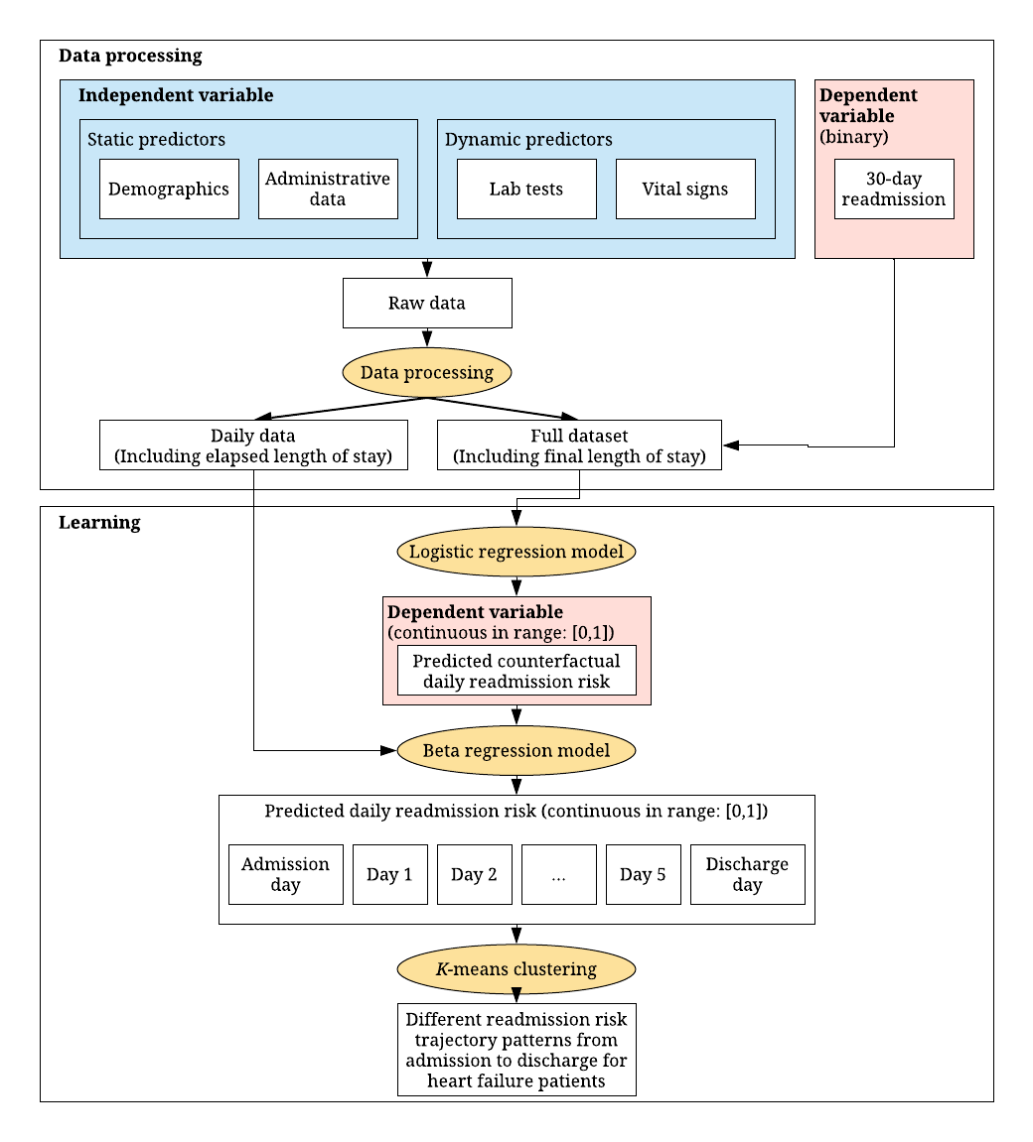
Flowchart of our modeling process. The rectangular boxes represent data input and output. The yellow oval boxes represent the models. Independent and dependent variables for each model are colored in blue and red, respectively.

Detailed steps of the entire modeling process are shown in Figure 1. The detailed steps summarizing the entire modeling continuum train the predictive models, learn patient groups by trajectory, and identify predictors that differentiate these patient groups are summarized in Multimedia Appendix 2.

We conducted all of our analysis using Python (version 2.7) and R (version 3.4.1).

## Results

### Setting and Data

In our sample, we observed 534 patient encounters by 454 unique patients that included 164 patients who experienced multiple encounters. In total, 30-day readmission occurred in 107 (107/534, 20.0%) of these encounters. Characteristics of the patient cohort stratified by readmission outcome may be seen in Table 1.

### Supervised 2-Stage Prediction Model

Logistic regression with backward selection identified an optimal set of 57 predictors for our first-stage encounter-level prediction model. These optimal predictors are displayed in Table 2 from a total pool listed in Multimedia Appendix 3. Dynamic clinical predictors measuring laboratory results and vital signs, specifically potassium, sodium, BUN, hemoglobin, and diastolic blood pressure, demonstrated the most predictive value. Overall, the number of medication orders for digoxin, number of measurements of peripheral capillary oxygen saturation (SpO2), and number of hemodialysis performed were negatively associated with readmission risk. The number of measurements of sodium and number of times mechanical ventilation was used were both positively associated with readmission risk. Discharge disposition was a significant administrative predictor. The patients who were discharged to a nursing facility compared with the non-nursing facility had a much higher readmission risk (odds ratio of 10.59). Overall, the out-of-sample AUC of the first-stage encounter-level logistic regression model (Table 2) was 0.73 (SD 0.08). The R-squared value for the second-stage daily prediction beta regression model was 0.88.

**Table 2:** Summary of significant predictors for the logistic regression model with backward feature elimination.

Predictor			Coefficient	Odds ratio (95% CI)	*P* value
Potassium: normalized index of minimal potassium			−2.15	0.12 (−3.27 to −1.03)	<.001
SpO2^a^: number of measurements of SpO2			−0.07	0.93 (−0.11 to −0.03)	.001
**Hemoglobin**					
	First value−last value		.56	1.75 (0.20 to 0.92)	.002
	Normalized index of maximal hemoglobin		1.63	5.14 (0.30 to 2.98)	.02
Digitalis glycosides: number of medication order			−0.67	0.51 (−1.11 to −0.23)	.003
Blood urea nitrogen: first value−last value			−0.04	0.96 (−0.07 to −0.01)	.004
Discharge disposition: nursing Facility			2.36	10.59 (0.62 to 4.10)	.008
**Number of procedures**					
		Mechanical ventilation	0.08	1.08 (0.02 to 0.14)	.008
		Hemodialysis	−0.41	0.67 (−0.80 to −0.01)	.04
**Diastolic blood pressure**					
		First value−last value	−0.02	0.98 (−0.04 to −0.01)	.008
		Number of measurements	0.05	1.05 (0.01 to 0.09)	.02
		Average value of last 3 diastolic blood pressure	−0.05	0.96 (−0.09 to −0.004)	.03
Sodium: normalized index of minimal sodium			−1.21	0.30 (−2.25 to −0.18)	.02
Respiratory rate: normalized index of minimal respiratory rate			1.12	3.07 (0.10 to 2.15)	.03
ZIP code: 210XX			0.77	2.17 (0.05 to 1.50)	.04

^a^SpO2: peripheral capillary oxygen saturation.

### Unsupervised Clustering of Readmission Risk Trajectories

**Figure 2:**
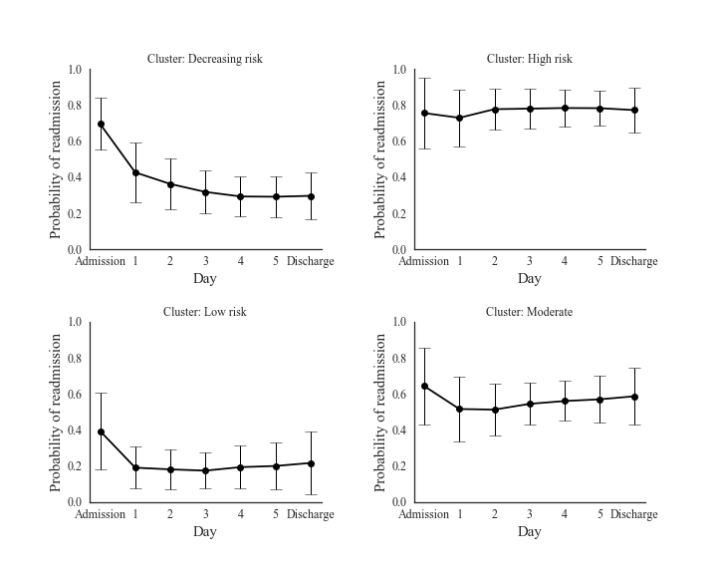
Daily readmission probabilities for four clusters of patients are shown from admission to discharge. The error bars represent one standard deviation on each side of the expected readmission probabilities.

A total of 4 distinct trajectories emerged from the unsupervised clustering analysis as seen in Figure 2. The *decreasing risk* group with 131 encounters (131/534, 24.5%) was unique in demonstrating appreciable change in risk over the hospitalization from admission (.69 probability of readmission) to discharge (.30). The remaining trajectory groups maintained a consistent readmission risk over time. This included the *high risk* group of 113 (113/534, 21.2%) encounters with average readmission risk maintained above 0.75 over their course of care. The *low risk* cluster with 113 (113/534, 21.2%) encounters were admitted with a relatively low average readmission risk of 0.39 that decreased to 0.21 at discharge. The *moderate risk* cluster had 177 (177/534, 33.1%) encounters, and its average readmission risk had a mean of 0.61.

Discriminant analyses demonstrated 18 predictors with similar characteristics across the 4 risk trajectory groups (Kruskal Wallis *P*<.001) as seen in Multimedia Appendix 4. The most discriminant predictors were laboratory measures (eg, potassium, hemoglobin, sodium, and ALT), diastolic blood pressure, and the number of past hospital visits. Large decreases in potassium and the minimum potassium level occurring later in the hospitalization was associated with decreasing readmission risk. Lower hemoglobin and sodium levels were associated with higher readmission risk. High variability, maximum, and average measures of diastolic blood pressure were associated with consistently low readmission risk, whereas low diastolic blood pressure measures were associated with patients whose readmission risk remained high during hospitalization. Finally, the number of past hospital visits was associated with higher readmission risks.

**Figure 3:**
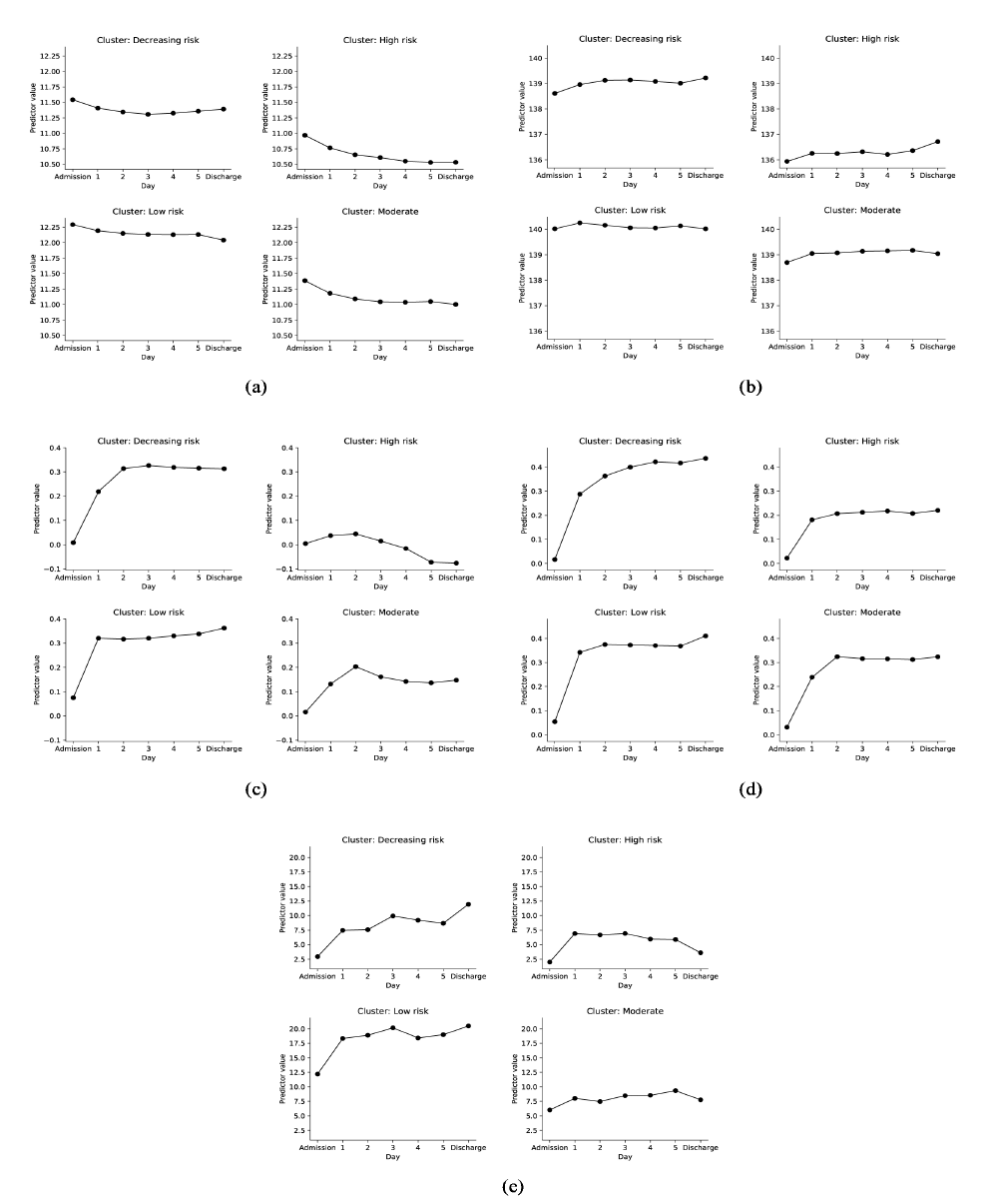
Change of discriminative predictors values over time from admission to discharge within each patient risk group. (a) Average value of last three hemoglobin measurements (gm/dL). (b) Average value of last three sodium measurements (mmol/L). (c) Decrease of potassium level from admission (mmol/L). (d) Normalized time of minimal potassium starting from admission. (e) Decrease of diastolic blood pressure level from admission (mmHg).

These discriminant predictors were further investigated by graphically displaying their temporal changes for each readmission trajectory group. Figure 3 depicts the primary discriminant features (eg, hemoglobin, sodium, potassium, and diastolic blood pressure) for each group. Associative patterns included the following: low and decreasing levels of hemoglobin was associated with higher readmission risk, low levels of sodium was associated with higher readmission risk, large decreases in potassium and minimum potassium levels occurring close to discharge was associated with lower readmission risk, and a large decrease of diastolic blood pressure from admission was associated with lower readmission risk.

## Discussion

### Principal Findings

By examining the changes of readmission risk over the course of a patient’s hospital stay, we discovered patient groups with distinct readmission risk trajectories. These groups may provide additional insight about discriminant predictors that can be undetected using traditional static prediction models. A portion of our findings were consistent with previous research on readmission prediction for patients with heart failure:

- The predictive performance (AUC) for our dynamic modeling approach was 0.73 (SD 0.08), which was relatively high but within the range (0.55-0.76 [3-8]) of performance reported in other studies that applied a static modeling approach for patients with heart failure.
- Number of previous hospitalizations and decreases in hemoglobin indicating anemia have similarly been associated with increased readmission risk [5,24-34].
- Nguyen et al found that vital sign instability at discharge was associated with increased risk-adjusted 30-day mortality and readmission rates [15]. Vital sign instability means abnormal values of vital signs. For instance, higher average hemoglobin measurements near discharge demonstrating stability was linked to lower readmission risk in our prediction model, similar to the findings from the study by Nguyen et al for general readmission [15].

However, there were some differences in our model findings not present in the literature to our knowledge. First, we found that dynamic clinical predictors measuring laboratory results and vital signs had the most value in predicting readmission risk and discriminating trajectories. This was contrary to the report by Hamill et al [6] of no incremental benefit of mixing clinical data with administrative claims data for readmission risk prediction. Specifically, higher levels of hemoglobin, a larger decrease in potassium, and diastolic blood pressure from admission to discharge indicate a lower readmission risk. Potassium changes during hospitalization of patients with heart failure are iatrogenic, that is, decreased by using intravenous diuretics and increased by repletion with oral or intravenous potassium supplementation. Therefore, it is not an intrinsic factor that affects heart failure risk. However, it can still serve as a surrogate indicator of heart failure readmission risk. A larger decrease in diastolic blood pressure probably reflects the condition of patients with diastolic heart failure who have increased diastolic blood pressure at admission [35]. Second, being different from past readmission prediction models that are static and only predict at discharge time [4-7], our model dynamically predicts readmission risk and facilitates informed interventions throughout patients’ stay. Finally, using our dynamical prediction model, we identified 4 different readmission risk trajectory patterns over patients’ stay. To our knowledge, our study is the first study that investigated the readmission risk trajectories over patient’s stay for patients with heart failure.

Overall, these findings support assessments that may be useful in better predicting readmission risk over the course of a patient’s stay. They include the following: (1) recognizing high readmission risks associated with patients who have been hospitalized frequently in the past, (2) abnormally low hemoglobin that is either consistent throughout the stay or drops near discharge is associated with higher risk of readmission, (3) abnormally low sodium is associated with high readmission risk, (4) abnormally high diastolic blood pressure late in the hospital stay or no considerable decreases (<4 mmHg) from admission to discharge may indicate higher readmission risk, (5) abnormally high potassium levels near discharge may indicate high risk of readmission, and (6) a minimal potassium occurring closer to discharge is associated with decreasing risk of readmission.

### Limitations

There were several limitations in our study data and methods that should be considered when interpreting results. First, clustering of time-dependent trends in readmission used up to 7 time points (admission, 5 days, and discharge) for each encounter. This approach was advantageous in enabling comparisons of readmission risk profiles across patients but limited in its ability to compare short-stay patients (eg, <5 days) with longer-stay patients (>5 days). Ideally, we would perform clustering analysis controlling for the entire duration of each patient’s stay. However, we believe that our 7–time point approach yielded results that would not qualitatively change as readmission risk trends vary less toward the end of the hospitalization, particularly for longer-stay patients. Second, owing to the limited sample size, we treated each encounter in our first-stage model as independent. When the same patient has multiple encounters, as seen in our data, this assumption may be contravened. This becomes particularly important to avoid bias in out-of-sample performance evaluation (ie, training and test sets). However, we determined that treating each patient encounter independently was analogous to how this predictive tool could be run in real time. We also further mitigated risks of bias by performing 5-fold cross-validation. Third, we used mean imputation to impute missing data in features. The disadvantage of mean imputation is that it reduces the variance of the imputed features and, as a result, the discriminability of that feature. A more comprehensive method to impute missing data may improve the prediction accuracy. Finally, evaluating predictive performance of our second-stage predictive model presented challenges methodologically. We were unable to observe the counterfactual readmission outcomes (readmission outcomes if the patient were discharged earlier than their actual discharge time). Using the last day’s outcomes to estimate the predictive performance of the second-stage model is an incomplete estimate, as we ignore the predictive performance on all days before the day of discharge. Therefore, we used a pseudo R-squared value to estimate the predictive performance of the second-stage model.

### Broad Impact

We applied supervised machine learning using a novel 2-stage approach to construct a 30-day readmission prediction model that yields daily predictions for patients with heart failure over time. We estimated the unknown counterfactual daily readmission risk using an encounter-level logistic regression model (first stage). A beta regression model using predictors engineered from EHR data accumulated to the prediction time (ie, each day) was fit to form the daily readmission risk prediction model (second stage). Investigating dynamic trends in readmission risk over time via unsupervised clustering, we uncovered for distinct groups based on readmission risk trajectory: decreasing, high, moderate, and low. The clinical features distinguishing these groups (eg, hemoglobin, sodium, potassium, and diastolic blood pressure) may shed light on new indicators of readmission for patients with heart failure and the isolated effects of the index hospitalization. Our ultimate goal is to use the important features and our model to develop a clinical tool for dynamically predicting readmission for patients with heart failure and target patients with high readmission risk. The clinical tool is aimed to show readmission risk for each patient and also the corresponding factors that are associated with high or low readmission risk. This study provides a first step in the development of such a tool by proposing a dynamic prediction model for readmission risk of patients with heart failure.

## Appendix

Multimedia Appendix 1 Example of the daily data (patient-day data) used in the second stage model.

Multimedia Appendix 2 Algorithm 1. Steps for the entire modeling process.

Multimedia Appendix 3 Relevant categories of features (57 total) selected using logistic regression and backward feature elimination.

Multimedia Appendix 4 Summary of discriminative predictors for each patient group.

## Abbreviations

ALT: alanine transaminase
AST: aspartate transaminase
AUC: area under curve
BUN: blood urea nitrogen
EHR: electronic health record
proBNP: pro B-type natriuretic peptide
