# Supplementary material for: Readmission Risk Trajectories for Patients With Heart Failure Using a Dynamic Prediction Approach: Retrospective Study
Source: JMIR Med Inform. 2019 Sep 16;7(4):e14756. doi: 10.2196/14756 (PMC6781727; doi:10.2196/14756)
Supplement: Multimedia Appendix 1 [file medinform_v7i3e14756_app1.pdf]

Example of the daily data (patient-day data) used in the second stage model.

Encounter ID	Age	Minimal hemoglobin (gm/dl)	⋯	Elapsed Length of stay (days)	Estimated counterfactual daily readmission risk
1	91	13.8	⋯	0	0.12
1	91	13.8	⋯	1	0.09
1	91	11.3	⋯	2	0.07
1	91	10.8	⋯	3	0.06
1	91	10.6	⋯	4	0.04
1	91	10.2	⋯	5	0.02
1	91	10.2	⋯	5.5	0.01
2	60	10.6	⋯	0	0.16
2	60	10.5	⋯	1	0.18
2	60	9.9	⋯	1.7	0.18
⋮	⋮	⋮	⋮	⋮	⋮
534	80	14.3	⋯	0	0.85
534	80	12.9	⋯	1	0.79
534	80	12.9	⋯	1.9	0.75
