# Supplementary material for: Readmission Risk Trajectories for Patients With Heart Failure Using a Dynamic Prediction Approach: Retrospective Study
Source: JMIR Med Inform. 2019 Sep 16;7(4):e14756. doi: 10.2196/14756 (PMC6781727; doi:10.2196/14756)
Supplement: Multimedia Appendix 2 [file medinform_v7i3e14756_app2.pdf]

**Algorithm 1. Steps for the entire modeling process**

1. Obtain the full dataset in the format of **Error! Reference source not found.**
2. Fit the full dataset with an optimal set of selected predictors into a logistic regression model and obtain the estimated model parameter $\widehat{\boldsymbol{\beta}}$ using maximum likelihood estimation.

   Logistic regression model: $p(y = 1|X, \boldsymbol{\beta}) = \frac{1}{1+ e^{-\boldsymbol{\beta}^T X}}$

   where $y$ is the '30-day readmission' outcome, $X$ is the vector of predictors in the full dataset (row in **Error! Reference source not found.** excluding encounter ID and readmission outcome)
3. Obtain the daily data in the format of **Error! Not a valid bookmark self-reference.**
4. Estimate the counterfactual daily readmission risk for each patient-day (row of **Error! Not a valid bookmark self-reference.**) using the trained logistic regression from the first stage and daily data in **Error! Not a valid bookmark self-reference.**:

   $$p\left(\text{counterfactual daily readmission risk}\middle|X, \widehat{\boldsymbol{\beta}}\right) = \frac{1}{1+ e^{-\widehat{\boldsymbol{\beta}}^T X}}$$

   where $X$ is the vector of predictors in the daily data (row in **Error! Not a valid bookmark self-reference.** excluding encounter ID and readmission risk outcome)
5. Fit the daily data in **Error! Not a valid bookmark self-reference.** into a beta regression model and obtain the estimated model parameters $\widehat{\boldsymbol{\gamma}}, \widehat{\phi}$ using maximum likelihood estimation

   Beta regression model: $p(y|\mu, \phi) = \frac{\Gamma(\phi)}{\Gamma(\mu\phi)\Gamma((1-\mu)\phi)} y^{\mu\phi-1}(1-y)^{(1-\mu)\phi-1}$

   where $\mu = \frac{1}{1+ e^{-\gamma^T X}}$ , $y$ is the estimated counterfactual daily readmission risk outcome from step 2, and $X$ is a vector of predictors in the daily data (row in **Error! Not a valid bookmark self-reference.** excluding encounter ID and readmission risk outcome)
6. Assemble the time series of daily readmission risk predicted by the beta regression model trained in step 5 for each patient
7. Apply the K-means clustering algorithm to cluster patients into groups based on their respective time series trajectories of daily readmission risk
8. Apply Kruskal-Wallis test to identify discriminative predictors associated with the different patient groups identified in step 7

9. Analyze how the above discriminative predictors change with time and are associated with different readmission risk patterns.
