# Supplementary material for: Readmission Risk Trajectories for Patients With Heart Failure Using a Dynamic Prediction Approach: Retrospective Study
Source: JMIR Med Inform. 2019 Sep 16;7(4):e14756. doi: 10.2196/14756 (PMC6781727; doi:10.2196/14756)
Supplement: Multimedia Appendix 3 [file medinform_v7i3e14756_app3.pdf]

Relevant categories of features (57 total) selected using logistic regression and backward feature elimination

Categories	Features
**Administrative**	Discharge destination: Charlestown Community Inc (SNF MD ONLY)
	Discharge destination: Ellicott City Health & Rehabilitation Center (SNF MD ONLY)
	Discharge destination Future Care Pineview (SNF MD ONLY)
	Discharge destination other Maryland skilled nursing facility (SNF MD ONLY)
	Discharge disposition: Intermediate Care Facility
	Discharge disposition: nursing facility
	Discharge disposition: rehab unit - other acute hospitals
	Insurance
	Number of index diagnose
	ZIP 21044
	Count of prior hospital visits
**Alanine transaminase**	Average absolute change ALT (Units/L)
	Average of last 3 ALT (Units/L)
	Maximal ALT (Units/L)
**Aspartate transaminase**	Average absolute change AST (Units/L)
	Average AST (Units/L)
	First minus last AST (Units/L)
	Minimal AST (Units/L)
**Peripheral capillary oxygen saturation**	Average of last 3 SPO2
	Number of measurement SPO2
**Sodium**	Average absolute change sodium (mmol/L)
	Average sodium (mmol/L)

	Minimal sodium (mmol/L)
	Normalized location of minimal sodium (mmol/L)
	Number of measurement sodium (mmol/L)
**Creatinine**	First minus last creatinine (mg/dL)
**Blood urea nitrogen**	First minus last BUN (mg/dL)
**PROBNP**	Normalized location of maximal PROBNP (pg/mL)
	Number of measurement PROBNP (pg/mL)
**Hemoglobin**	First minus last hemoglobin (gm/dL)
	Normalized location of maximal hemoglobin (gm/dL)
**Systolic blood pressure**	Average absolute change systolic blood pressure
	Average of last 3 systolic blood pressure
	Average systolic blood pressure
	Maximal systolic blood pressure
	Standard deviation of systolic blood pressure
**Diastolic blood pressure**	Average of last 3 diastolic blood pressure
	First minus last diastolic blood pressure
	Maximal diastolic blood pressure
	Normalized location of maximal diastolic blood pressure
	Number of measurement diastolic blood pressure
	Standard deviation of diastolic blood pressure
**Potassium**	Average absolute change potassium (mmol/L)
	Average potassium (mmol/L)
	Normalized location of minimal potassium (mmol/L)
**Respiratory rate**	Normalized location of minimal respiratory rate
**Weight**	Average weight
	First minus last weight
	Maximal weight
	Minimal weight
	Number of measurement weight

**Medication order**	Number of medication order: antihypertensives, angiotensin receptor antagonist
	Number of medication order: digitalis glycosides
**Procedure**	Number of procedure: BIPAP
	Number of procedure: hemodialysis
	Number of procedure: mechanical ventilation
	Number of procedure: patient may use own CPAP
