# Supplementary material for: Readmission Risk Trajectories for Patients With Heart Failure Using a Dynamic Prediction Approach: Retrospective Study
Source: JMIR Med Inform. 2019 Sep 16;7(4):e14756. doi: 10.2196/14756 (PMC6781727; doi:10.2196/14756)
Supplement: Multimedia Appendix 4 [file medinform_v7i3e14756_app4.pdf]

Summary of discriminative predictors for each patient group.

Predictor	Boxplot
**Diastolic blood pressure**	
Standard deviation (mmHg)	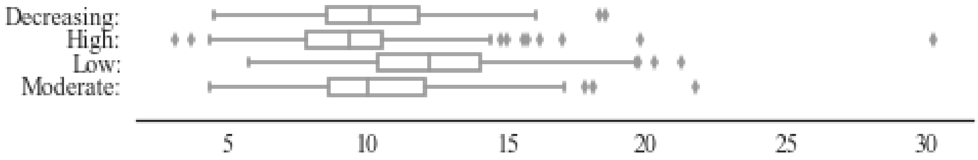
First value–last value (mmHg)	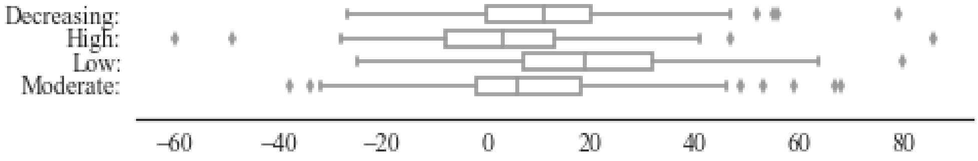
Maximal value (mmHg):	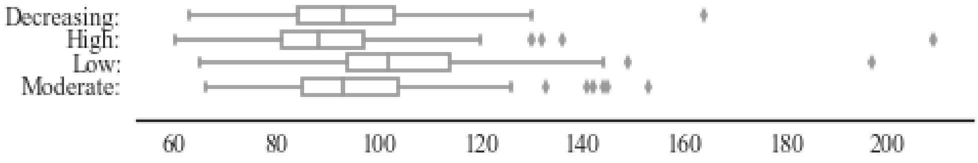
Average value (mmHg)	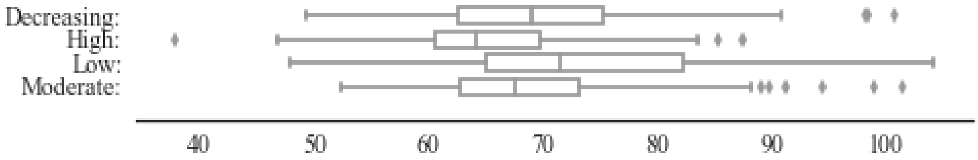
Average absolute change (mmHg)	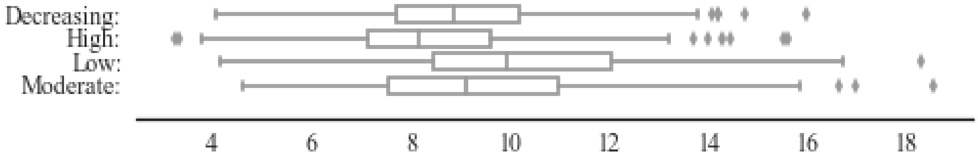
**Potassium**	
Normalized index of minimal value	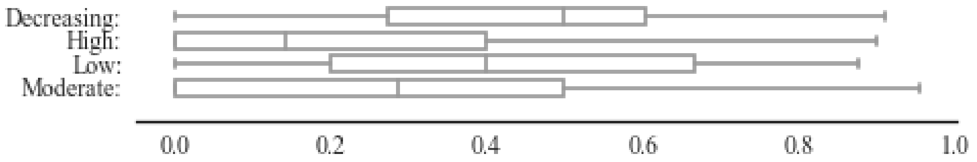
First value—last value (mmol/L)	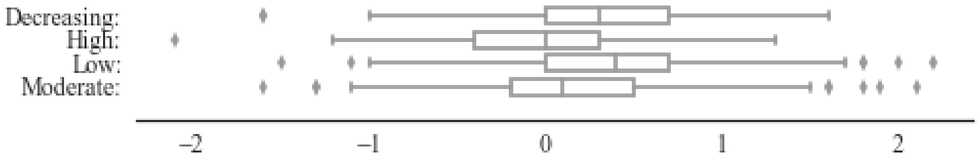

	Average absolute change (mmol/L)	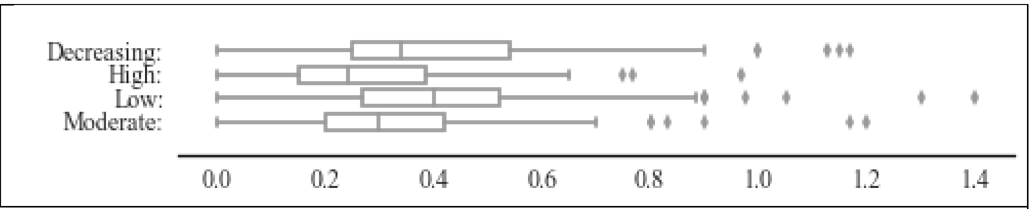
**Sodium**		
	Average value (mmol/L)	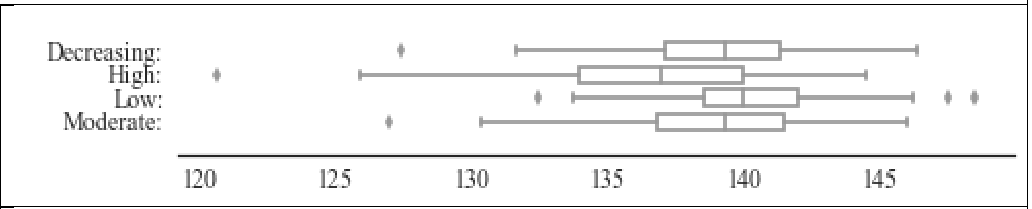
	Maximal value (mmol/L)	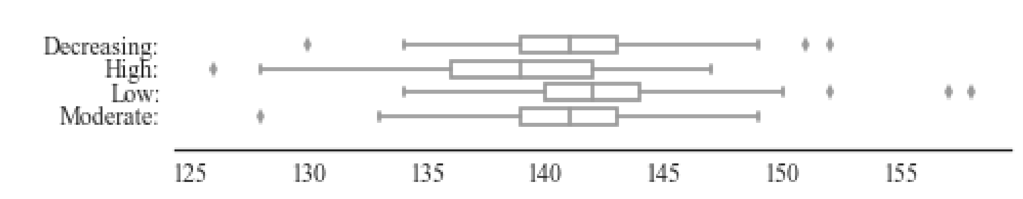
	Average of last 3 values (mmol/L)	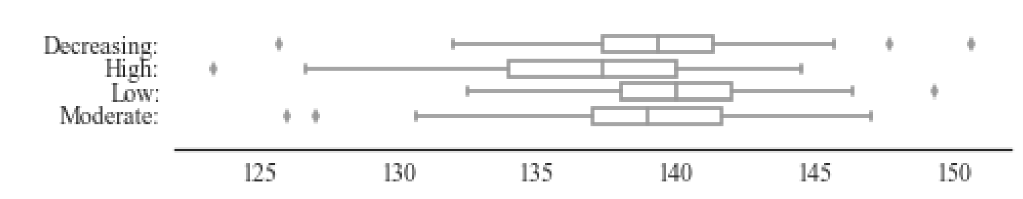
	Minimal value (mmol/L)	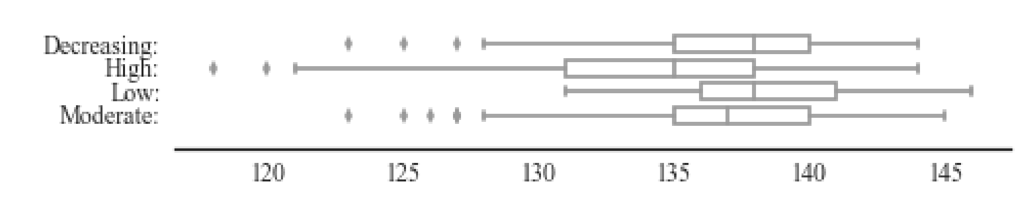
**Hemoglobin**		
	Minimal value (gm/dL)	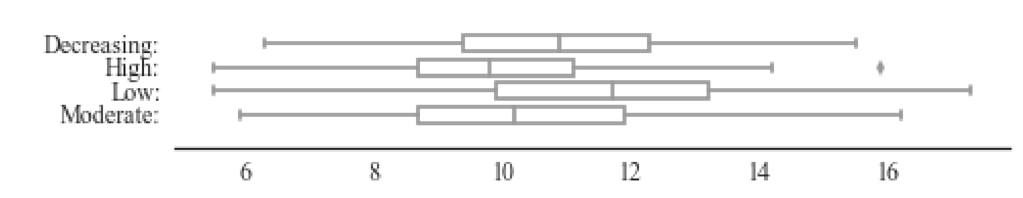
	Average value (gm/dL)	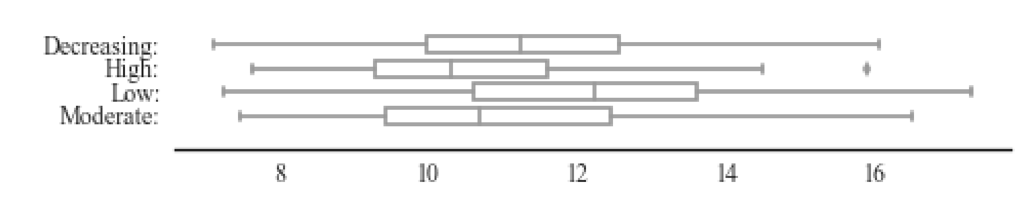
	Average of last 3 values (gm/dL)	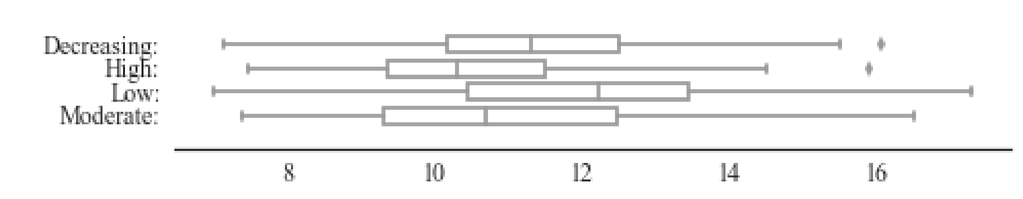

	Maximal value (gm/dL)	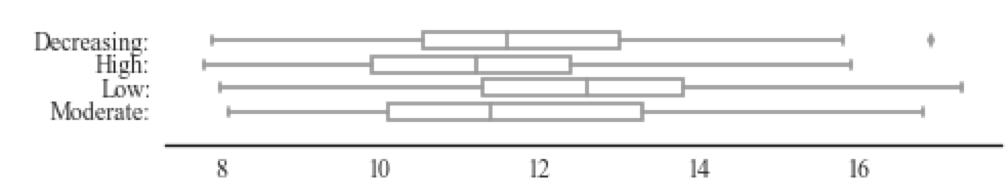
Alanine transaminase : minimal value (units/L)		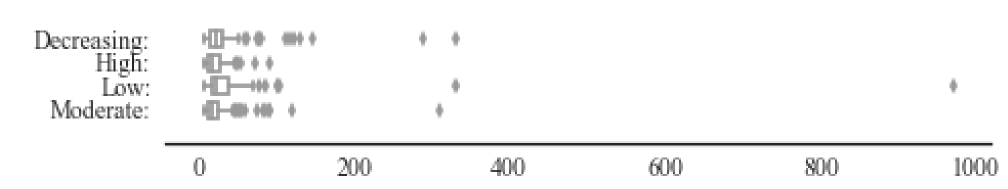
Number of past [illegible]		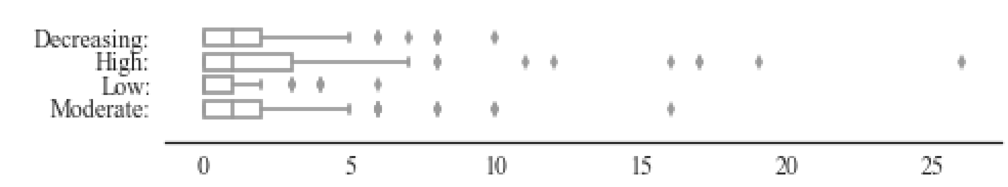
